# Spectral Convolution Feature-Based SPD Matrix Representation for Signal Detection Using a Deep Neural Network

**DOI:** 10.3390/e22090949

**Published:** 2020-08-28

**Authors:** Jiangyi Wang, Min Liu, Xinwu Zeng, Xiaoqiang Hua

**Affiliations:** 1 College of Meteorology and Oceanography, National University of Defence Technology, Changsha 410073, China; wjy05460731@163.com; 2 College of Computer, National University of Defence Technology, Changsha 410073, China; liumin_nudt@163.com

**Keywords:** SPD matrix learning, local features, deep neural networks, signal detection

## Abstract

Convolutional neural networks have powerful performances in many visual tasks because of their hierarchical structures and powerful feature extraction capabilities. SPD (symmetric positive definition) matrix is paid attention to in visual classification, because it has excellent ability to learn proper statistical representation and distinguish samples with different information. In this paper, a deep neural network signal detection method based on spectral convolution features is proposed. In this method, local features extracted from convolutional neural network are used to construct the SPD matrix, and a deep learning algorithm for the SPD matrix is used to detect target signals. Feature maps extracted by two kinds of convolutional neural network models are applied in this study. Based on this method, signal detection has become a binary classification problem of signals in samples. In order to prove the availability and superiority of this method, simulated and semi-physical simulated data sets are used. The results show that, under low SCR (signal-to-clutter ratio), compared with the spectral signal detection method based on the deep neural network, this method can obtain a gain of 0.5–2 dB on simulated data sets and semi-physical simulated data sets.

## 1. Introduction

In recent years, convolutional neural networks have shown excellent performance in visual tasks. Starting from AlexNet [1], many successful convolutional neural network models have been developed, such as VGG [2], GoogLeNet [3], ResNet [4], and DenseNet [5]. With the help of hierarchical convolution kernel and nonlinear computation, deep neural networks can extract more discriminative local features for visual representations. Literature [6] proves that the convolution feature is a set of local features related to objects, all of which together describe the visual characteristics of objects. Rich feature descriptions draw researchers’ great interest in deep convolution features. To enhance feature learning, a lot of aggregation operations based on deep convolution features are proposed, such as max pooling [7], cross-dimensional pooling [8], sum pooling [6], and bilinear pooling [9]. These operations are in high-dimensional Euclidean spaces.

For the past few years, the SPD (symmetric positive definite) matrix has drawn considerable attention because of its powerful representation ability. Based on non-Euclidean Riemannian geometric properties, the SPD matrix is more suitable for capturing the desired data distribution properties. The SPD matrix manifold is widely used in medical imaging [10], sonar signal processing [11], radar signal processing [12], face recognition [13,14], action recognition [15,16], object or image classification [17,18], and transfer learning [19]. The nonlinear distribution of the SPD matrix in the Riemannian manifold can be measured on a non-Euclidean scale, based on the geodetic distance.

Transforming convolution features into SPD matrices is a problem of converting first-order data into second-order statistic information. Gaussian distribution and covariance matrix are widely used SPD matrix representations that transform first-order features into second-order statistical information [20,21]. However, the dimension of the convolution feature is often higher than the number of features. In this case, both the Gaussian distribution and the covariance matrix are singular matrices, and the data distribution does not conform to the manifold property of the SPD matrix. It is inappropriate to exploit Riemannian metrics to distinguish the information difference. To solve this problem, a small perturbation can be added to the covariance [22]. Besides, kernel functions can characterize nonlinear relationships of data. In [23], positive definite kernel based on Gaussian radial basis function is defined on manifold, and Euclidean space algorithms, such as support vector machine (SVM) and principal component analysis (PCA) are extended to Riemannian manifold with the help of the proposed positive definite kernel, based on Gaussian radial basis function. With the advent of deep matrix learning [24,25,26], literature [27] proposes a deep SPD matrix learning model, which exploits RBF kernel function to aggregate convolution features into SPD matrices. Their ultimate goal is to convert the SPD matrix from a Riemannian manifold to another more distinctive manifold.

Based on the above empirical observations, we introduce a method to transform spectral convolution features into SPD matrices through an RBF kernel function, and then detect the target signal using a deep SPD matrix learning network. Two modules are included in this method. The first module uses a nonlinear function to transform convolution features of the spectrum into SPD matrices. All generated SPD matrices become processing objects for the second module. The second module utilizes SPDnet [25]. It is a deep matrix learning network consisting of a SPD matrix transformation layer, SPD matrix nonlinear processing layer, log-Euclidean projection layer, and fully connected (FC) layers. SPD matrices become more compact and discriminative after being passed through the SPD matrix transformation layer and SPD matrix nonlinear processing layer. After using the log-Euclidean metric, the difference in geodetic distance between SPD matrices generated by different samples will be projected into the Euclidean space, and the dichotomization problem based on the Riemannian manifold will be solved by the FC layer based on the Euclidean space. The convolution features of the spectrogram correspond to the information annotation of the spectrogram in advance. Theoretically, SPD matrices based on pre-processed samples can have more significant differences. The experimental results show that compared with the signal detection method based on spectrum, our method can obtain a gain of 0.5–2 dB on the simulation data set, and can obtain a gain of 0.5–1 dB on the semi-physical simulation data set.

The rest is in the following order: In Section 2, we elucidate the processing approach of input samples by the convolution neural network and some relevant contents of convolution features. In Section 3, we introduce the nonlinear kernel function and the RBF kernel function used in our paper. In Section 4, we provide the spectrum SPD matrix learning method on the basis of a deep network for signal detection. In Section 5, we will first evaluate the performance of the method, by using the simulation data set based on K-distribution clutter as disturbance, and explore the influence of its hyperparameters, and then compare the performance by using the semi-physical simulation data set on the basis of the measured sea clutter as disturbance. In the end, we provide a conclusion in Section 6.

## 2. Acquisition of Convolution Features

The convolution features are derived from the convolutional neural network. It originates from the neocognitron proposed in 1980 [28], and the first model LeNet appeared in 1998 [29]. Since 2012, the research of convolutional neural networks has gained significant achievement. The main advantage of convolutional neural networks compared to their predecessors is that it can automatically detect the important features of input data without any human supervision. For example, in image classification, the convolutional neural network can automatically learn the differences between different categories of images. Additionally, in semantic segmentation, through continuous iteration, the convolutional neural network can discover the features of different types of regions in the image, and identify different targets in the image according to the features learned.

**Figure 1:**
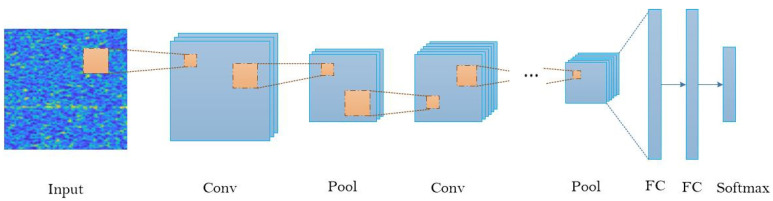
General flow diagram of the convolutional neural network. Although different network models differ, they all basically follow this paradigm.

Although models of the convolutional neural network vary widely, they all follow some similar principles. Figure 1 shows the general architecture of a convolutional neural network for a classification task.

When an image is fed to the convolutional neural network, it will be processed by a series of convolutional layers and pooling layers. The image is continuously reduced in dimension and finally vectorized into the full connection layers to get the decision result.

Figure 1 omits layers such as nonlinear layers, and normalized layers that perform particular calculations. They are usually immediately behind a convolutional layer and are not distributed throughout the network. Convolution and pooling are the main layers of a convolutional neural network. We mainly introduce the theoretical basis and process of image convolution features extracted by convolutional layers, and the role of pooling in convolution feature extraction.

**Figure 2:**
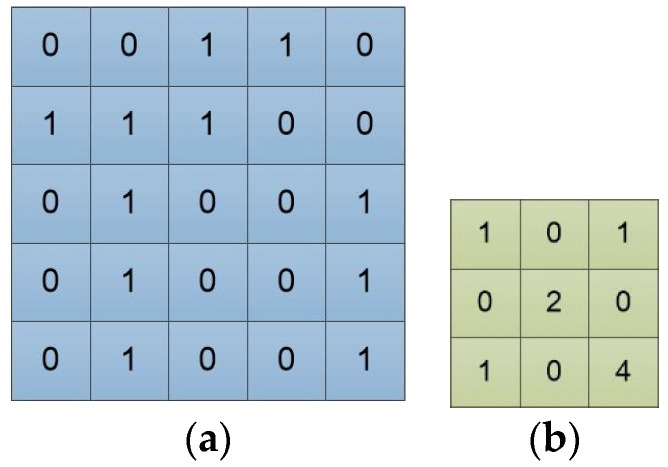
Input image and filter. (**a**) Input image converted to matrix array form; (**b**) The convolution filter/kernel, and in this paper, it is called the convolution filter.

An image is represented by a matrix of integers, and each pixel corresponds to each element value in the matrix. A monochromatic image is a two-dimensional matrix, while an RGB image is a three-dimensional matrix. Figure 2a is an input example, and can be viewed as a single-channel image. The “0” and “1” represent pixel values. Figure 2b is a 3 × 3 convolution filter. Each of its element values represents the weight of the position. The feature extraction ability of the convolution filter is closely related to its size and weights.

**Figure 3:**
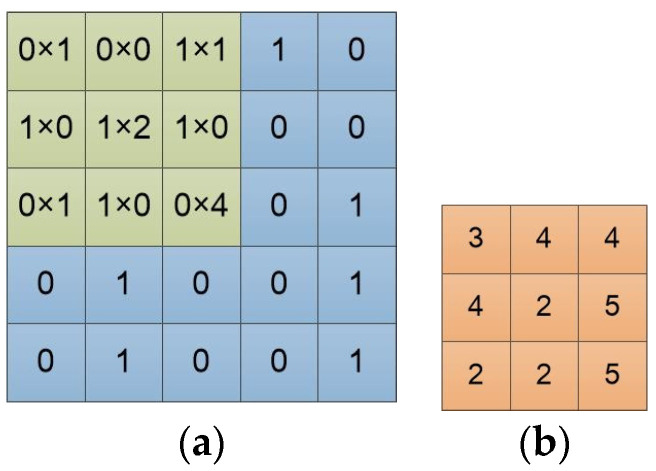
The processing and result of the input image by a convolution filter. (**a**) The computational details of the convolution filter on the input image; (**b**) The computed result of the convolution filter on the input image, and this result is directly fed to the next convolutional layer, or is vectorized firstly and then fed to the following fully connected layer.

In mathematics, convolution is an operation on two functions that produces a third function expressing how the shape of one is modified by the other. The convolution operation in the convolutional neural network adds each element of the image (or feature map) to its local neighbors, weighted by the convolution filter, to produce a new feature map. The computational relationship between the convolution filter and the image in Figure 2 is shown in Figure 3a. We slide the convolution filter over the input data. As Figure 3b shows, a local multiplication is performed at every location, and we sum the result onto the feature map.

The weighted sum between the convolution filter and the image enhances the effects of some local features of the image and suppresses the rest. The convolution features are enhancements of some features of the original image. Different convolution filters can extract different features, and these filters are learnable. Typically, the output feature maps are enhanced with specific features. The feature map will be used as the input of the next convolutional filter, to generate a more abstract feature map with a lower dimension.

**Figure 4:**
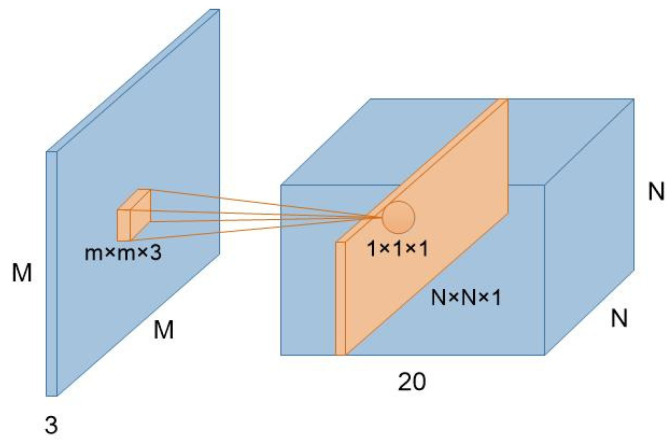
The detailed flowchart of the convolution layer to the input image.

**Figure 5:**
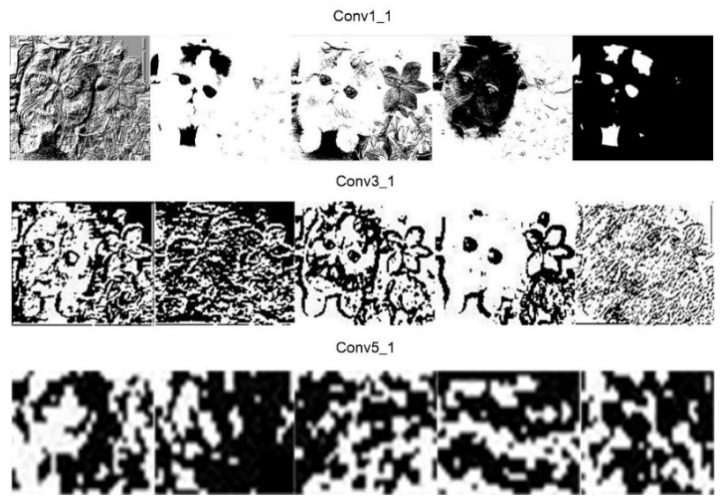
The convolution of VGG19 with different depth extracts convolution features of the input image. As layers get deeper, the convolution features of feature maps become more and more abstract. Different convolution filters generate different feature maps, which can be seen in the figure.

An image is represented as a three-dimensional matrix, with dimensions of height, width, and depth (color channels). So, the convolution filter has a depth dimension, and it covers the entire depth of its input. It is also important to note that multiple convolutions on input are performed, each using a different filter and resulting in a distinct feature map. The final output of the convolution layer is the superposition of all feature maps. Figure 4 shows the convolution computation of an input image by a convolution filter. There are twenty convolution filters to deal with the input, respectively, and the generated feature map has a depth of 20. Figure 5 is some feature maps output by different convolution layers of VGG19. From the Conv1_1 layer to the Conv5_1 layer, the depth of the network is increasing, the extracted convolution feature is more and more abstract, the number of feature maps generated by the same layer is increasing, and the dimension is getting lower and lower.

The pooling layer also plays a role in the generation of convolution features. This section provides a brief introduction to the most commonly used pooling: max pooling.

**Figure 6:**
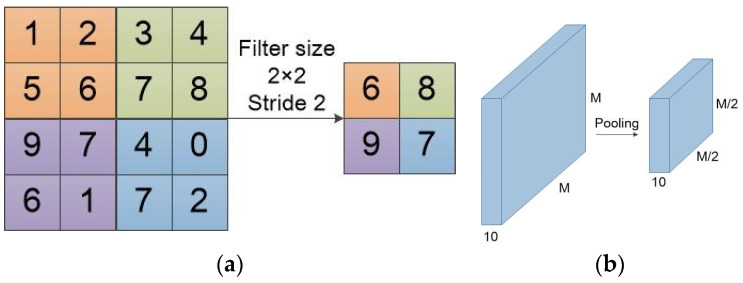
Schematic diagram of the calculation process of a max pooling layer. (**a**) The calculation of a feature map by max pooling; (**b**) The detailed dimension reduction of pooling on the input.

As shown in Figure 6, the calculation process of a max pooling is similar to convolution. However, the max pooling calculation reserves the maximum value in the calculation region. The output of max pooling is the image that retains the most obvious convolution feature. Max pooling reduces the dimension of feature maps, but does not increase or decrease the number of feature maps.

## 3. Construction of SPD Matrix Based on Convolution Feature

Although the covariance matrix is used to model the features and has acquired gratifying results, two issues still remain ineluctable. One is that the covariance matrix may be singular. This situation usually occurs when the dimension of local features is larger than the number of local features extracted from the image area. The other one is that the covariance matrix cannot evaluate the nonlinear correlation of features. It is not conducive to the modeling of convolution features. To settle the above two issues, we exploit the nonlinear kernel function to construct the SPD matrix.

We set $X\in {\mathbb{R}}^{C\times H\times W}$ as the set of all convolution features of one layer in the convolutional neural network. C is the number of feature maps, H and W are the height and width of the feature map, respectively. $x_{i}\in {\mathbb{R}}^{C}$ is the i-th local feature and $f_{i}\in {\mathbb{R}}^{H\times W}$ is the i-th feature map. The modeling of nonlinear kernel function revolves around $f_{i}$.

Elements in the SPD matrix $K\in {\mathbb{R}}^{C\times C}$ generated by nonlinear kernel function can be defined as:

(1) $$K_{ij}=Κ\left(f_{i},f_{j}\right)=\langle \varphi \left(f_{i}\right),\varphi \left(f_{j}\right)\rangle ,$$

where φ(·) is the implicit mapping, 〈·,·〉 is the pairwise inner product.

In this paper, we exploit the RBF kernel function to construct SPD matrices. It is expressed as:

(2) $$Κ\left(f_{i},f_{j}\right)=exp\left(-\|f_{i}-f_{j}\|{}^{2}/2\sigma^{2}\right),$$

where σ is the mean Euclidean distances of all feature maps, ‖ · ‖ is the Euclidean norm.

The RBF kernel function can guarantee the positive definiteness of the matrix. The proof is as follows:

To simplify the proof, we rewrite Formula (2) as:

(3) $$Κ\left(f_{i},f_{j}\right)=exp\left(-\alpha \|f_{i}-f_{j}\|{}^{2}\right),$$

where α is used to denote $1/2\sigma^{2}$ for the purpose of simplification.

The Fourier transform convention of the Formula (3) is:

(4) $$\kappa \left(\omega \right)={\left(2\pi /\alpha \right)}^{n/2}\int_{R^{n}}e^{i\omega f_{i}}e^{-i\omega f_{j}}e^{-\omega^{2}/\left(2\alpha \right)}d\omega ,$$

Then, we prove that the quadratic form of the kernel matrix K is always greater than 0. Set $c=\left(c_{1},\ldots ,c_{M}\right)\in {\mathbb{R}}^{C\times 1}$, and it is an arbitrary non-zero vector. The quadratic expression is obtained as:

$$Q=c^{T}Kc=\overset{C}{\underset{j=1}{\sum }}\overset{C}{\underset{k=1}{\sum }}c_{j}c_{k}exp\left(-\alpha \|f_{i}-f_{j}\|{}^{2}\right)$$

(5) $$=\overset{C}{\underset{j=1}{\sum }}\overset{C}{\underset{k=1}{\sum }}c_{j}c_{k}{\left(2\pi /\alpha \right)}^{n/2}\int_{R^{n}}e^{i\omega f_{i}}e^{-i\omega f_{j}}e^{-\|\omega {\|}^{2}/\left(2\alpha \right)}d\omega$$

$$={\left(2\pi /\alpha \right)}^{n/2}\int_{R^{n}}e^{-\|\omega {\|}^{2}/\left(2\alpha \right)}\|\overset{C}{\underset{j=1}{\sum }}c_{j}e^{-i\omega f_{j}}\|{}^{2}d\omega ,$$

In Formula (5), $e^{-\|\omega {\|}^{2}/\left(2\alpha \right)}$ is positive and continuous, and the quadratic form is 0 on the condition that $\sum_{j=1}^{C}c_{j}e^{-i\omega f_{j}}=0$. However, the complex exponentials $e^{-i\omega f_{1}},\ldots ,e^{-i\omega f_{M}}$ are linear independence. Thus, the quadratic form is always greater than 0, and matrix K is positive definite.

**Figure 7:**
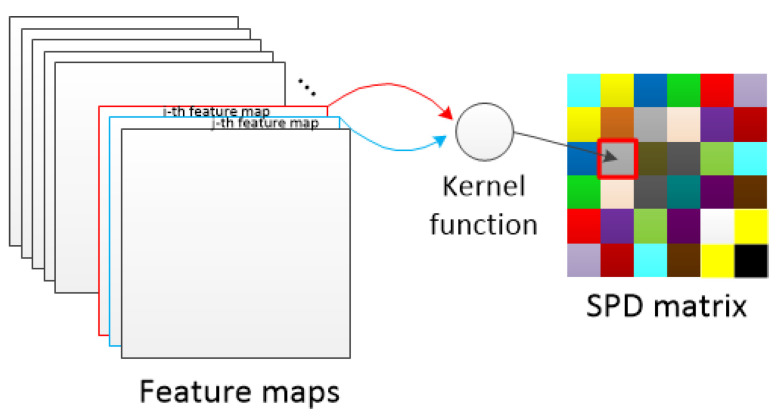
The process of converting a set of feature maps into an SPD matrix. An SPD matrix is constructed from all feature maps in the same layer of a spectrogram sample. Each element on the SPD matrix reflects the correlation between the two feature maps.

The above is the whole process of the positive definiteness; proof of the RBF kernel function. The use of the RBF kernel function to construct the SPD matrix is essentially a correlation calculation between feature maps. Figure 7 shows the calculation process of the correlation operation.

Formula (2) reveals the nonlinear relationship between convolution features. It can be calculated faster by means of matrix operations through proper modification.

Convolution features $X\in {\mathbb{R}}^{C\times H\times W}$ can be reshaped to a matrix $M\in {\mathbb{R}}^{C\times N}$ and N=H×W. Each feature map $f_{i}\in {\mathbb{R}}^{H\times W}$ can be expanded into vector form in a certain order $f_{i}\in {\mathbb{R}}^{1\times N}$. Moreover, the $\|f_{i}-f_{j}\|{}^{2}$ is equivalent to:

(6) $$\|f_{i}-f_{j}\|{}^{2}=f_{i}f_{i}^{T}-2f_{i}f_{j}^{T}+f_{j}f_{j}^{T},$$

Three terms to the right of Formula y can be computed by matrix multiplication:

$$f_{i}f_{i}^{T}=1{\left(M◦M\right)}^{T}$$

(7) $$f_{j}f_{j}^{T}=\left(M◦M\right)1^{T}$$

$$f_{i}f_{j}^{T}=MM^{T},$$

where ◦ stands for the Hadamard product and $1\in {\mathbb{R}}^{C\times N}$ is the matrix whose elements are all “1”.

We name $f_{i}f_{i}^{T}$ to X1, name $f_{j}f_{j}^{T}$ to X2 and name $f_{i}f_{j}^{T}$ to X3. formula (2) is modified to the following:

(8) $$Κ=exp\left(-\left(X1+X2-2X3\right)/2\sigma^{2}\right),$$

where exp(A) is the exponential operation to each element in the matrix A.

In this paper, we use VGG19 and GoogLeNet-V3 to extract convolution features. For VGG19, we use feature maps for its pool1, pool2, and pool3 output. These three layers are located in layer 5, layer 10, and layer 19 of VGG19, and their depths are 64, 128, and 256, respectively. This also means that SPD matrices they build are 64 × 64, 128 × 128, 256 × 256, respectively. For GoogLeNet-V3, we use feature maps output by its Maxpool_5a_3 × 3 layer, which is located in layer 6 of GoogLeNet-V3 and has a depth of 192.

## 4. SPDnet: A Deep Matrix Learning Method

SPDnet is characterized by embedding bilinear mapping (BiMap) and eigenvalue rectification (ReEig) into the algorithm, in the form of layers of the deep learning network. Their effect on SPD matrices is equivalent to the convolution layer and nonlinear computing layer of the convolution neural network, both of which aim to make objects to be processed more compact and discriminative.

The computation expression for a BiMap layer to perform bilinear mapping is

(9) $$X_{k}=W_{k}X_{k-1}W_{k}^{T},$$

where $X_{k-1}\in Sym_{d_{k-1}}^{+}$ is the input matrix of the k-th layer, $W_{k}\in {\mathbb{R}}_{*}^{+},(d_{k}<d_{k-1})$ is a transformation matrix, which is row full-rank, and $X_{k}\in Sym_{d_{k}}^{+}$ is the output matrix.

The computation expression for a ReEig layer to perform eigenvalue rectification is

(10) $$X_{k}=U_{k-1}\max\left(\varepsilon I,\Sigma_{k-1}\right)U_{k-1}^{T},$$

where $U_{k-1}$ and $\Sigma_{k-1}$ are achieved by singular value decomposition, $X_{k-1}=U_{k-1}\Sigma_{k-1}V_{k-1}^{T}$, ε indicates a threshold, I indicates an identity matrix, and $\max\left(\varepsilon I,\Sigma_{k-1}\right)$ means retaining the elements in $\Sigma_{k-1}$ that are greater than ε and replacing the rest with ε.

Exploiting singular value decomposition instead of eigenvalue decomposition to analyze the SPD matrix is an efficient approximation method. The computational speed of singular value decomposition is faster than that of eigenvalue decomposition. Similar to the eigenvalues, singular values are arranged from large to small in the diagonal matrix generated by the decomposition. In most cases, the sum of the first 10% singular values occupies around 99% of the sum of all singular values. Based on the square matrix property of the SPD matrix, the approximate reconstruction by the ReEig layer is reasonable.

The LogEig layer is used to perform Riemannian calculation on SPD matrices generated after multiple bilinear mappings and eigenvalue correction. The log-Euclidean Riemannian metric endows the SPD matrices Riemannian manifold with a Lie group structure, and the SPD matrix manifold is reduced to a flat space. The log-Euclidean metric is described in detail below.

The space of C×C SPD matrices can form a Riemannian manifold $S_{+}^{C}$. It has a globally defined differential structure and has the possibility to define the derivatives of the curves on the SPD matrix manifold. The tangent space $T_{S_{1}}S_{+}^{C}$ of point $S_{1}$ on the manifold can be constructed by exploiting logarithm map $log_{S_{1}}:S_{+}^{C}\to T_{S_{1}}S_{+}^{C}\left(S_{1}\in S_{+}^{C}\right)$, and an inner product $\langle ,\rangle_{S_{1}}$. The family of inner products on Riemannian manifold’s all tangent spaces is called the Riemannian metric of the manifold. The geodesic distance between two points $S_{1}$, $S_{2}$ on the SPD matrix manifold can be expressed as $\langle log_{S_{1}}\left(S_{2}\right),log_{S_{1}}\left(S_{2}\right)\rangle_{S_{1}}$ in the framework of Riemannian metric.

The log-Euclidean metric of the SPD matrix manifold corresponds to the Euclidean metric in the SPD matrix logarithmic domain. Thus, the scalar product between $T_{1}$ and $T_{2}$ in the tangent space at the point **S** is

(11) $$\langle T_{1},T_{2}\rangle {}_{S}=\langle D_{S}log.T_{1},D_{S}log.T_{2}\rangle ,$$

where $D_{S}log.T$ is the directional derivative of the matrix logarithm at **S** along with **T**.

The logarithmic and exponential maps related to the metric can be expressed as

(12) $$\begin{array}{c} log_{S_{1}}\left(S_{2}\right)=D_{\log\left(S_{1}\right)}exp.\left(\log\left(S_{2}\right)-\log\left(S_{1}\right)\right)\\ exp_{S_{1}}\left(T_{2}\right)=exp\left(\log\left(S_{1}\right)+D_{S_{1}}log.T_{2}\right), \end{array}$$

where $D_{\log\left(S_{1}\right)}exp.={\left(D_{S}log.\right)}^{-1}$ roots in the differentiation of the equality log◦exp=I, and I is the identity matrix.

From Formulas (11) and (12), the geodesic distance obtained through log-Euclidean metric is

(13) $$D_{le}\left(S_{1},S_{2}\right)=\langle log_{S_{1}}\left(S_{2}\right),log_{S_{1}}\left(S_{2}\right)\rangle_{S_{1}}=\|\log\left(S_{1}\right)-\log\left(S_{2}\right){\|}_{F}^{2},$$

Formula (13) is to the Euclidean distance in the logarithmic domain. Under the log-Euclidean metric, the distance between any two points on SPD matrix manifold is acquired through propagating by translation the scalar product in the tangent space at the identity matrix. The space of SPD matrices is reduced to a flat Riemannian space with the help of the log-Euclidean.

The disadvantage of the log-Euclidean metric is that it is not an affine invariant metric, which leads to the fact that it cannot fully reflect the geodesic distance between two points on the Riemannian manifold. However, the form of the log-Euclidean metric has low computational complexity, and it is widely used in Riemannian manifold-based algorithms. For more details about the log-Euclidean metric, please refer to the literature [30].

The computation expression for the LogEig layer is

(14) $$X_{k}=log\left(X_{k-1}\right)=U_{k-1}log\left(\Sigma_{k-1}\right)U_{k-1}^{T},$$

where $X_{k-1}=U_{k-1}\Sigma_{k-1}U_{k-1}^{T}$ and $log\left(\sum_{k-1}\right)$ is the diagonal matrix of eigenvalue logarithms. After the treatment of LogEig layer, the linear classification method on the basis of Euclidean space can be used to perform tasks.

We mainly exploit the following SPDnet model with 8 layers: $X_{0}\to f_{BM}^{1}\to f_{RE}^{2}\to f_{BM}^{3}\to f_{RE}^{4}\to f_{BM}^{5}\to f_{LE}^{6}\to f_{FC}^{7}\to f_{soft}^{8}$, where $X_{0},f_{BM},f_{RE},f_{LE},f_{FC},f_{soft}$ is the input SPD matrix to the model, BiMap layer, ReEig layer, LogEig layer, fully-connected layer, and softmax log-loss layer, respectively.

## 5. Results

### 5.1. Experimental Analysis of Simulation Data

In this part, we made a comparision with two signal detection methods on the basis of the deep neural network: deep network based on time-frequency spectrum for signal detection and deep SPD matrix learning network for signal detection based on spectrum convolution feature. The data originated from a simulated signal data set, and the simulated clutter based on K-distribution is used as interference. K-distribution is a widely accepted sea clutter simulation model. Compared with Rayleigh distribution, lognormal distribution and Weibull distribution, it considers the correlation between echo pulses, and better fits the amplitude distribution of sea clutter.The probability density of K-distribution follows the following formula:

(15) $$f\left(x\right)=\frac{2}{a\Gamma \left(v\right)}{\left(\frac{x}{2a}\right)}^{v}K_{v-1}\left(\frac{x}{a}\right),$$

where $K_{v-1}\left(\cdot \right)$ indicates the second modified Bessel function of order v−1, Γ(·) indicates the Gamma function, a indicates the scale parameter to represent the mean value of clutter, which is set as 1 in this paper, v indicates the shape parameter to affect the shape of the distribution curve, which is set as 1 in this paper.

Target signal is generated by simulation. Its driving vector is

(16) $$p=\frac{1}{\sqrt{N}}{\left[1,\exp\left(j2\pi f_{d}\right),\ldots ,\exp\left(j2\pi \left(N-1\right)f_{d}\right)\right]}^{T},$$

where N indicates the number of pulses of the signal and is set as 2048, $f_{d}$ indicates the normalized Doppler frequency, set to 0.15.

The definition of normalized Doppler frequency is

(17) $$f_{d}=\frac{F_{d}}{f_{s}}=\frac{vf_{c}}{cf_{s}},$$

where $F_{d}=\frac{vf_{c}}{c}$ indicates the Doppler frequency of the simulated target, v indicates the relative velocity of the target, set to 5 m/s, c indicates the speed of the electromagnetic wave and is $3\times 10^{8}$ m/s, $f_{c}$ indicates the carrier frequency of the radar, set to 9.39 GHz, $f_{s}$ indicates the pulse repetition rate of the radar, set to 1000 Hz.

**Figure 8:**
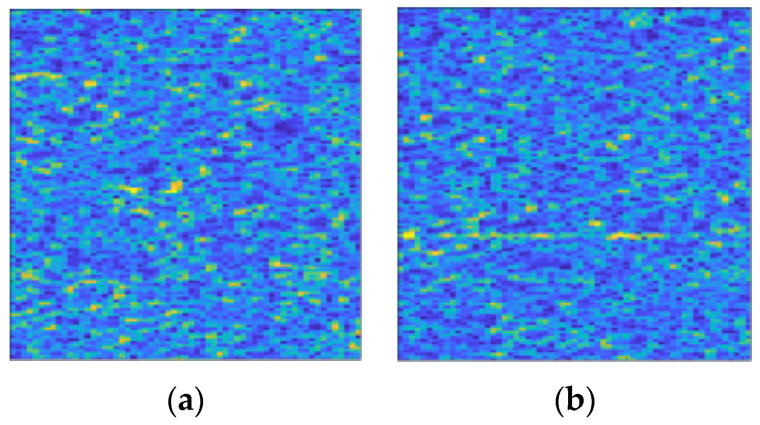
Time-frequency spectrum color maps used in this study. K-distribution clutter is exploited. (**a**) The sample containing only clutter; (**b**) The sample containing signal interfered by clutter. Its SCR is −15 dB.

There are 11,000 samples with merely clutter and no target signal. The SCR of clutter interfered samples with the target signal is distributed from −5 dB to −20 dB at 1 dB intervals, with 4000 samples for each SCR. The time-frequency spectrum in the form of color map involved in this section is displayed in Figure 8.

#### 5.1.1. Comparison with Convolutional Neural Networks

**Figure 9:**
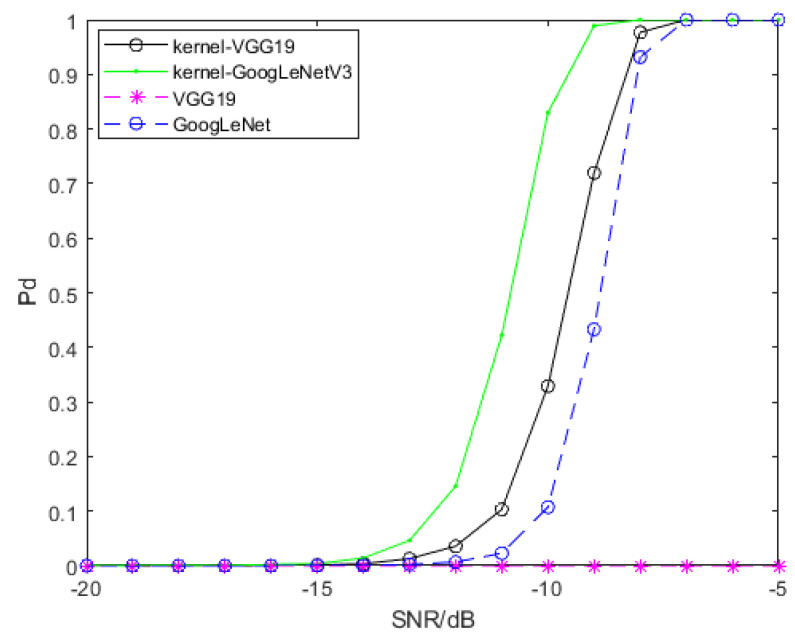
Detection probability of models involved in this section. To ensure that the false alarm probability of all models does not exceed $10^{-4}$, the SCR of the signal samples interfered by clutter used in training is −5 dB. “kernel-VGG19” on behalf of the SPD matrix network trained by SPD matrices based on pool2 convolution features of VGG19, and “kernel-GoogLeNetV3” on behalf of the SPD matrix network trained by SPD matrices based on convolution features of GoogLeNetV3.

For the purpose of signal detection, SPDnet is exploited to process SPD matrices generated by convolution features, and GoogLeNet and VGG19 are exploited to process time-frequency spectra. Featuremaps of time-frequency spectra are generated and ourput by Tensorflow 1.8, and then they are processed by RBF kernel function to generate SPD matrices. We use transfer learning to adapt GoogLeNet and VGG19 to our signal detection problem, and retrain the SPDnet. The samples used for training all models are samples that only contain clutter and clutter interfered signal samples with SCR of −5 dB, −10 dB and −15 dB. Every training session, we use 1000 pure clutter samples and 1000 clutter disturbed signal samples, and the SCR of the latter is the same. The remaining samples are used to evaluate the detection performance of each model. Model training will have some preset parameters, which will not be updated with iteration, and we name them hyperparameters. The hyperparameters involved in this paper and their set values are as follows: the batch size, set as 20; the learning rate, set as 0.001; the weight decay, set as 0.0005; the epoch for SPDnet, set as 500; the epoch for GoogLeNet and VGG19, set as 800. All models have been fully trained. We trained SPDnet on an i7-8565U CPU, while GoogLeNet and VGG19 were trained on an Nvidia GeForce GTX 1080 Ti GPU. The detection probability of the above models is shown in Figure 9. The SCR of signal samples used for training is −5 dB.

Figure 9 shows that two SPD matrix learning networks based on convolution features have advantages in detection problem. On the whole, the detection performance of two SPD matrix networks based on convolution features is better than that of GoogLeNet, and VGG19 cannot detect the target signal. The gain of the SPD matrix network trained by SPD matrices based on pool2 convolution features of VGG19 relative to GoogLeNet is about 0.6 dB when the detection probability of both models reaches 70%, and the gain of the SPD matrix network trained by SPD matrices based on the convolution features of GoogLeNetV3 relative to GoogLeNet is 2 dB when the detection probability of both models reaches 80%.

Table 1 is the comparision of the false alarm probability about different models involved in this paper. The false alarm probability of them is not greater than $10^{-4}$ under the circumstance of the SCR is −5 dB. With the decrease of SCR, the false alarm probability of them increases, but that of two SPD matrix network models increases greatly.

#### 5.1.2. The Effect of Hyperparameters

**Figure 10:**
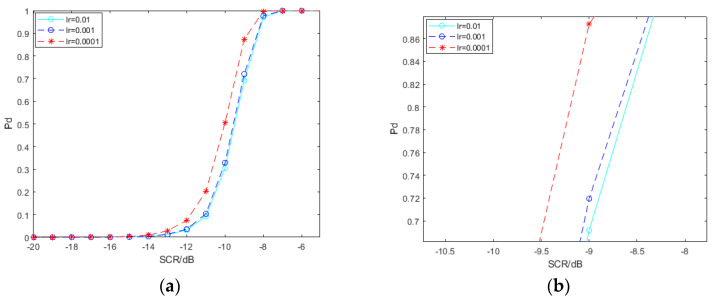
Detection probability under different learning rates. (**b**) is the local amplification of (**a**).

**Figure 11:**
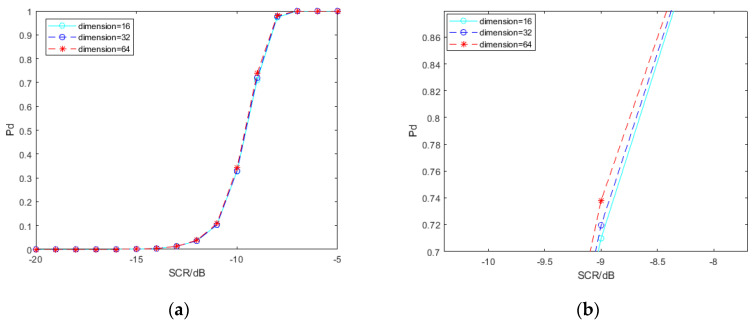
Detection probability under different dimensions. (**b**) is the local amplification of (**a**).

**Figure 12:**
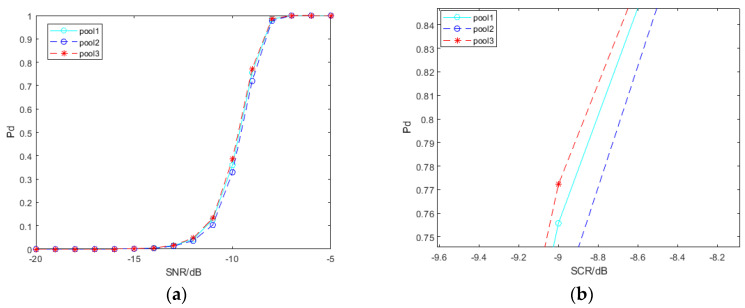
Detection probability curves under SPD matrices constructed by different convolution layers. (**a**) shows the general trends; (**b**) shows the differences when the detection probability is around 80%.

The learning effect of deep neural networks is influenced by hyperparameters. We explore the impact of the learning rate, the output layer of convolution features and the dimension of the SPD matrix before entering the LogEig layer, on the performance of the spectral convolution feature-based SPD matrix learning method, with deep learning for signal detection. In order to simplify the work, we merely use the SPD matrix network based on pool2 convolution features of VGG19. The signal samples interfered by clutter used by the network models involved in Figure 10, Figure 11 and Figure 12 are all −5 dB.

The detection probability of the SPD matrix learning network trained by SPD matrices based on pool2 convolution features of VGG19 under different learning rates is shown in Figure 10. The results show that the low learning rate is beneficial to the improvement of detection probability. The SPD matrix learning method for signal detection which the learning rate is set as 0.0001 gets a gain of about 0.5 dB compared with others under the circumstance of the detection probability; 80%. In the backpropagation algorithm of a neural network, the learning rate is the step length of each iteration to find the local optimum. A high learning rate may skip the optimum. If the training time is sufficient, a small learning rate helps find the local optimum.

In this paper, “dimension” refers to the size of the SPD matrix before entering the LogEig layer. The SPD matrix will be more compact and discriminative through bilinear mapping and eigenvalue rectification. However, the reduction of matrix size is bound to cause some features to be lost theoretically. The results shown in Figure 11 confirm this conjecture. The detection probability is slightly optimized as the dimension increases. It is important to note that the overall effect is small.

From Figure 5, the deeper the network layer is, the more abstract the convolution feature is extracted. In theory, there are differences in the convolution features extracted from different layers to construct the SPD matrix. We select the convolution features of VGG19 output at three layers to construct SPD matrices: pool1, pool2, and pool3. The influence of convolution features generated by different layers is shown in Figure 12. As illustrated, the detection performance of SPD matrices based on convolution features of pool1 and pool3 is better than that based on convolution features of pool2. This may be related to the convolution features and characteristics of SPD matrices.

On the one hand, the deeper the layer is, the more abstract the convolution feature is extracted, which may lead to a loss of information for signal detection. The convolution features extracted at the shallow level retain more information, but in this case, there are fewer feature maps, and the generated SPD matrix dimension is smaller. On the other hand, although the convolution features extracted at a deeper level are more abstract, there are more feature maps and more comprehensive descriptions of samples, which can build the SPD matrix with a larger dimension.

The false alarm probability of above models when changing learning rate, dimension and the output layer of convolution features is shown in Table 2, Table 3 and Table 4. In general, the false alarm probability increases rapidly with the decrease of SCR. Only when SCR is −5 dB, false alarm probability is within the allowed range.

### 5.2. Experimental Analysis of Semi-Physical Simulation Data

**Figure 13:**
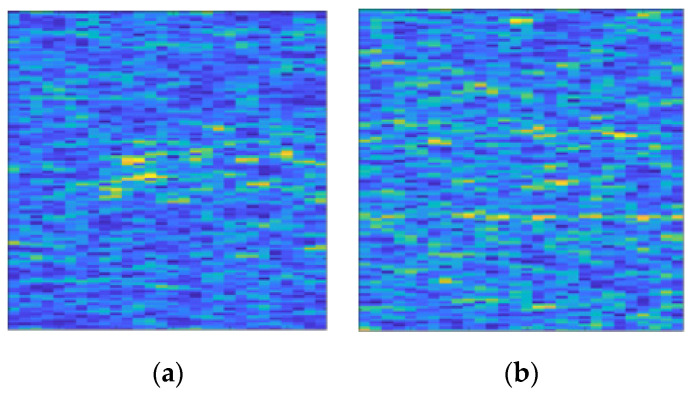
Time-frequency spectrum color maps used in this study. IPIX sea clutter is exploited. (**a**) The sample containing only clutter; (**b**) The sample containing signal interfered by clutter. Its SCR is −15 dB.

In this section, we select and use part of the IPIX (Ice Multiparameter Imaging X-Band) lake radar echo data as the clutter exerting interference [31]. Table 5 and Table 6 are the data files used and their basic parameters. Due to the data having no clear target information, we added one target signal. The number of pulses per sample is 1000, and other parameters are consistent with Section 5.1. The time-frequency spectrum in the form of color map involved in this section is shown in Figure 13.

The false alarm probability of different models is shown in Table 7. With the decrease of SCR, the false alarm probability of all models increases rapidly, which may be related to the uneven distribution of real sea clutter. The false alarm probability of the two models of convolutional neural increases more slowly than that of the two models based on SPD matrix learning.

**Figure 14:**
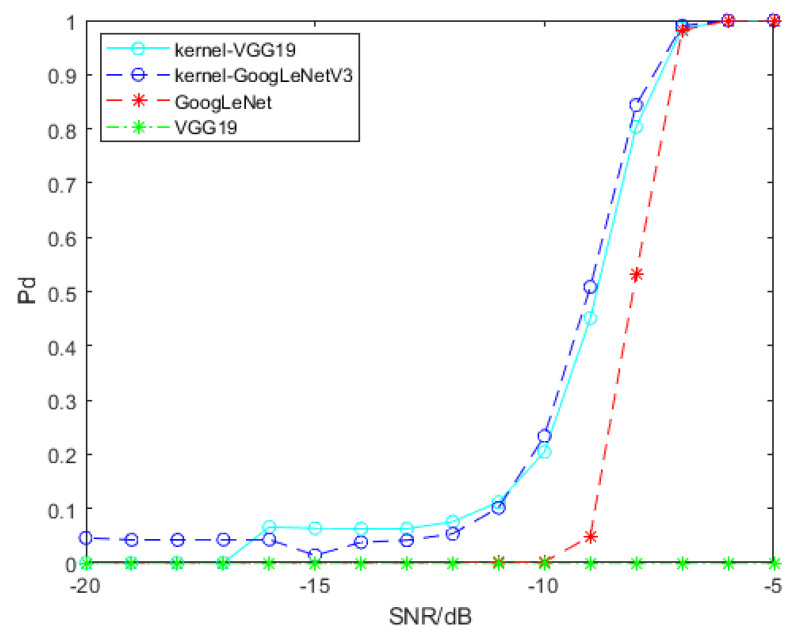
Detection probability of models in this section. The probability of false alarm is not greater than $10^{-4}$. “kernel-VGG19” is the SPD matrix learning network based on pool2 convolution features of VGG19, and “kernel-GoogLeNetV3” is the SPD matrix learning network based on the convolution features of GoogLeNetV3.

The detection property of the SPD matrix learning networks for signal detection and the convolutional neural networks is shown in Figure 14. Compared with Section 5.1, the detection property have changed significantly. The detection property of two SPD matrix learning networks on the basis of convolution features are better than that of GoogLeNet and VGG19. The advantage of two SPD matrix networks based on convolution features over GoogLeNet is obvious when SCR is low.

### 5.3. Complexity Analysis

In this section, we analyze the complexity of the involved models from two aspects: training time and size of space occupied by models. The comparison of training time and size of space occupied is shown in Table 8 and Table 9, respectively. Note that GPU is not used for training SPD matrix networks, as mentioned in Section 5.1.1. By comparison, it can be seen that our model is of low complexity.

## 6. Conclusions

We present a deep SPD matrix method for signal detection on the basis of spectral convolution features. Utilizing the SPD matrix obtained by transforming convolution features through the nonlinear kernel function and integrating with the deep SPD matrix learning network, a target signal under low SCR can be detected by the present method. The superiorities of this approach are that it combines the convolution features with the SPD matrix, converts original samples to the SPD matrix space, exploits its Riemannian manifold property to enhance the discriminability between different samples. Synchronously, the use of deep learning ameliorates the capacity of signal detection. A simulation date set with K-distribution clutter as interference and a semi-physical simulation data set with real sea clutter as interference are used by us to evaluate the performance of our method. The final results show that this method is effective, and it can obtain a gain of 0.5–2 dB on the simulation data set, and can obtain a gain of 0.5–1 dB on the semi-physical simulation data set. We explored the impacts of the hyperparameters associated with our method. It is inevitable that, due to the non-uniformity of interference in the real environment, our method may have poor detection ability. Our next goal is to enhance the anti-interference ability of our method and reduce the possibility of false alarms.

## Figures and Tables

**Table 1:** False alarm probability of different models (expressed as %).

	Model	VGG19-Kernel SPD Matrix Network	GoogLeNetV3-Kernel SPD Matrix Network	GoogLeNet with Time-Frequency Spectra	VGG19 with Time-Frequency Spectra
SCR (dB)					
−5		<0.01	<0.01	<0.01	<0.01
−10		0.97	0.1	<0.01	<0.01
−15		24.45	7.32	1.13	<0.01

**Table 2:** False alarm probability of different learning rates (expressed as %).

	Model	Learning Rate 0.01	Learning Rate 0.001	Learning Rate 0.0001
SCR (dB)				
−5		<0.01	<0.01	<0.01
−10		0.67	0.97	1.27
−15		5.35	24.45	25.57

**Table 3:** False alarm probability of different dimension (expressed as %).

	Model	16 Dimension	32 Dimension	64 Dimension
SCR (dB)				
−5		<0.01	<0.01	<0.01
−10		0.92	0.97	0.95
−15		22.58	24.45	20.95

**Table 4:** False alarm probability of SPD matrices generated by convolution features from different layers (expressed as %).

	Model	Pool1	Pool2	Pool3
SCR (dB)				
−5		<0.01	<0.01	<0.01
−10		0.92	0.97	0.97
−15		23.30	24.45	17.03

**Table 5:** The IPIX (Ice Multiparameter Imaging X-Band) radar data file used in this section.

Number	Name
1	19980223_171533_ANTSTEP
2	19980223_171811_ANTSTEP
3	19980223_172059_ANTSTEP
4	19980223_172410_ANTSTEP
5	19980223_172650_ANTSTEP
6	19980223_184853_ANTSTEP
7	19980223_185157_ANTSTEP

**Table 6:** Parameters of IPIX sea clutter signal.

Pulse Repetition Frequency	Carrier Frequency	The Length of the Pulse	Range Resolution	Polarization Mode
1000 Hz	9.39 GHz	60,000	3 m	HH

**Table 7:** The false alarm probability of models involved in this section (expressed as %).

	Model	VGG19-Kernel SPD Matrix Network	GoogLeNetV3-Kernel SPD Matrix Network	GoogLeNet with Time-Frequency Spectra	VGG19 with Time-Frequency Spectra
SCR (dB)					
−5		0.64	<0.01	<0.01	<0.01
−10		7.56	1.39	<0.01	<0.01
−15		28.17	1.64	0.15	<0.01

**Table 8:** Training time comparision of different models per 100 epochs (in Section 5.1.1).

	Model	VGG19-Kernel SPD Matrix Network	GoogLeNetV3-Kernel SPD Matrix Network	GoogLeNet with Time-Frequency Spectra	VGG19 with Time-Frequency Spectra
SCR (dB)					
−5		1618 s	1551 s	1599 s	3470 s
−10		1634 s	1567 s	1598 s	3483 s
−15		1629 s	1564 s	1624 s	3521 s

**Table 9:** Size of space occupied comparision of different models (in Section 5.1.1).

	Model	VGG19-Kernel SPD Matrix Network	GoogLeNetV3-Kernel SPD Matrix Network	GoogLeNet with Time-Frequency Spectra	VGG19 with Time-Frequency Spectra
SCR (dB)					
−5		163 KB	187 KB	22,217 KB	507,474 KB
−10		163 KB	187 KB	22,216 KB	507,473 KB
−15		162 KB	187 KB	22,216 KB	507,474 KB
